# Neural Oscillations in Aversively Motivated Behavior

**DOI:** 10.3389/fnbeh.2022.936036

**Published:** 2022-07-01

**Authors:** Michael S. Totty, Stephen Maren

**Affiliations:** Department of Psychological and Brain Sciences, Texas A&M Institute for Neuroscience, Texas A&M University, College Station, TX, United States

**Keywords:** oscillations, theta, gamma, fear, anxiety, hippocampus, medial prefrontal cortex, amygdala

## Abstract

Fear and anxiety-based disorders are highly debilitating and among the most prevalent psychiatric disorders. These disorders are associated with abnormal network oscillations in the brain, yet a comprehensive understanding of the role of network oscillations in the regulation of aversively motivated behavior is lacking. In this review, we examine the oscillatory correlates of fear and anxiety with a particular focus on rhythms in the theta and gamma-range. First, we describe neural oscillations and their link to neural function by detailing the role of well-studied theta and gamma rhythms to spatial and memory functions of the hippocampus. We then describe how theta and gamma oscillations act to synchronize brain structures to guide adaptive fear and anxiety-like behavior. In short, that hippocampal network oscillations act to integrate spatial information with motivationally salient information from the amygdala during states of anxiety before routing this information *via* theta oscillations to appropriate target regions, such as the prefrontal cortex. Moreover, theta and gamma oscillations develop in the amygdala and neocortical areas during the encoding of fear memories, and interregional synchronization reflects the retrieval of both recent and remotely encoded fear memories. Finally, we argue that the thalamic nucleus reuniens represents a key node synchronizing prefrontal-hippocampal theta dynamics for the retrieval of episodic extinction memories in the hippocampus.

## Introduction

Learning about potential danger is an adaptive process that is necessary for survival in all animals. Similarly, animals must be able to update these memories when threat is no longer present. In humans, this behavioral flexibility allows us to avoid potential harm while maintaining our daily lives and routines. However, traumatic experiences can lead to pathological levels of fear and behavioral rigidity in otherwise safe situations. For example, war veterans with posttraumatic stress disorders (PTSD) may re-experience traumatic memories and experience high levels of fear or panic when encountering triggering stimuli after they have returned home.

The dysregulation of neural circuits that guide adaptive learning and memory are thought to underlie trauma- and anxiety-related disorders (Milad and Quirk, 2012; Maren et al., 2013; Ressler et al., 2022). Activity within these brain circuits is coordinated by electrical oscillations that influence a range of neuronal processes to synchronize and modulate network function. Decades of research implicate oscillatory rhythms in both behavioral arousal and neural computation. Importantly, psychiatric disorders are associated with oscillatory abnormalities (Linkenkaer-Hansen et al., 2005; Uhlhaas and Singer, 2010; Fitzgerald and Watson, 2018). Therefore, a complete understanding of the neural circuits and oscillations underlying adaptive regulation of fear and anxiety is essential to developing and advancing therapeutic tools for psychiatric disorders.

In this review we detail recent advancements in our understanding of how neural oscillations regulate fear and anxiety. We first review literature demonstrating that neural oscillations are an intrinsic mechanism facilitating neural computation with a focus on well-studied hippocampal theta and gamma oscillations. Next, we describe how hippocampal-to-prefrontal theta synchronization underlies anxiety-like behavior. Finally, we discuss the current state of research on neural oscillations and aversive learning and memory with a focus on amygdala oscillations.

## What Are Neural Oscillations?

Neural oscillations are rhythmic changes in brain electrical activity that typically range in frequency from 1 to 250 Hz. Oscillations can be observed at various levels of brain organization, from the subthreshold membrane potential of single neurons to brain-wide fluctuations in low-frequency slow waves (Buzsáki and Draguhn, 2004). Neural oscillations can be recorded either with depth electrodes that detect extracellular local field potentials (LFPs) within the brain or with scalp electrodes that reveal the electroencephalogram (EEG) (Buzsáki et al., 2012; Reimann et al., 2013; Herreras, 2016). They are thought to underlie many cognitive functions, including attention and sensory perception (Ward, 2003; Schroeder and Lakatos, 2009; Obleser and Kayser, 2019) and learning and memory (Klimesch, 1996; Fell and Axmacher, 2011; Herweg et al., 2020). Specific functions that arise from or are facilitated by neural oscillations include information routing, cell assembly organization, synaptic plasticity, and interregional communication.

Multiple mechanisms underlie and influence oscillatory activity in the brain. Ultimately, neural oscillations are largely thought to emerge as a network property of reciprocally connected excitatory and inhibitory neurons (Whittington et al., 1995; Wang and Buzsáki, 1996; Gonzalez-Burgos and Lewis, 2008; Buzsáki and Wang, 2012). Within these networks, periods of precise inhibition are efficient for synchronizing large numbers of pyramidal cells and generating network oscillations, especially *via* fast-spiking gamma-aminobutyric acid (GABA) interneurons (Gonzalez-Burgos and Lewis, 2008). However, oscillations may additionally be generated by other mechanisms such as synaptic interactions between single-neuron oscillators (Llinás, 1988). Indeed, subsets of thalamic (Jahnsen and Llinás, 1984), hippocampal (Núñez et al., 1987; García-Muñoz et al., 1993; Butler and Paulsen, 2015), neocortical (Gutfreund et al., 1995; Hutcheon et al., 1996), and amygdalar (Pape et al., 1998) neurons possess ionic mechanisms that endow intrinsic resonance and oscillatory firing at one or more frequencies.

## Oscillatory Correlates of Hippocampal Computation

Our current understanding of the function of oscillations in neural coding comes in large part from investigations of oscillations commonly observed in the hippocampus (Vanderwolf, 1969; Stewart and Fox, 1990; Buzsáki et al., 1994; Vinogradova, 1995; Eichenbaum et al., 1999; Buzsáki, 2002; Buzsáki and Tingley, 2018). Thus, will we briefly summarize the dominant hippocampal oscillations and their role in hippocampal function to serve as a framework for considering the role of oscillations in other brain regions. It should be noted that there are various sleep-related neural oscillations that are critical for cognitive processes such as memory consolidation (Steriade et al., 1993; Datta, 1997, 2000; Buzsáki, 1998; Popa et al., 2010; Datta and O’Malley, 2013; Hutchison and Rathore, 2015; Boyce et al., 2016; Totty et al., 2017). This review, however, will primarily focus on theta and gamma oscillations during waking states. For a detailed review on sleep-related oscillations see Adamantidis et al. (2019).

### Theta Oscillations

One of the most prominent oscillations observed in the hippocampus of behaving rodents are theta oscillations commonly observed with peak frequencies ranging from 4 to 12 Hz. Type-1 theta oscillations are observed during locomotion, correlate to movement speed, are atropine-resistant, and are typically observed at higher theta frequencies (∼7–12 Hz) (Kramis et al., 1975; Bland, 1986; Buzsáki, 2002). Conversely, type-2 theta oscillations are commonly observed during immobility and are linked to anxiety and motivated behaviors; they are also atropine-sensitive and are typically observed at lower theta frequencies (∼4–7 Hz) (Sainsbury et al., 1987; Montoya et al., 1989; Mikulovic et al., 2018). These divergent characteristics suggest that Type-1 and Type-2 hippocampal theta oscillations are generated by distinct mechanisms and likely play different roles in motivated behaviors. The medial septum is thought to be the primary generator of hippocampal theta oscillations through long-range inhibitory pacemaker neurons (Stewart and Fox, 1990; Vinogradova, 1995; Hangya et al., 2009; Colgin, 2013). However, recent studies have found that divergent populations of glutamatergic and cholinergic medial septal neurons are distinctly associated with Type-1 and Type-2 hippocampal theta, respectively (Vandecasteele et al., 2014; Fuhrmann et al., 2015). Nonetheless, the hippocampus can generate theta oscillations without medial septum connections *in vitro* (Colgin, 2013), suggesting that there are multiple mechanisms for generating hippocampal theta rhythms.

Decades of work have revealed that theta oscillations are central to spatial and memory functions of the hippocampus. Specifically, there is substantial evidence that these oscillations temporally organize and bind participating neurons to form cognitive maps during spatial navigation and episodic memory (Eichenbaum et al., 1999; Buzsáki, 2005). For example, hippocampal “place” cells whose activity is determined by an animal’s spatial location fire in sequences that are synchronized to the theta rhythm (O’Keefe and Dostrovsky, 1971; Moser et al., 2015; Wang et al., 2015; Grieves et al., 2020). Although place cells increase their firing in a particular spatial location, they also fire sequentially at a preferential phase of the ongoing LFP theta oscillation, representing past, present, and future locations (Dragoi and Buzsáki, 2006). These temporally connected cells are referred to as cell assemblies. Thus, a single hippocampal theta cycle can package or “chunk” individual environmental locations and stimuli into *gestalt* spatial representations *via* cell assembly formation.

Not only do theta sequences represent past, present, and future locations, but choices between possible future trajectories and memories can be observed in alternating theta cycles (Johnson and Redish, 2007; Jezek et al., 2011; Kay et al., 2020; Robinson and Brandon, 2021). Thus, hippocampal theta oscillations enable the accurate encoding and retrieval of spatial environments, trajectories during navigation, and represent future decisions. However, the hippocampus does not merely process spatial information but also plays a critical role in the encoding and retrieval of episodic memory representations (Bird and Burgess, 2008; Nyhus and Curran, 2010; Lega et al., 2012; Eichenbaum and Cohen, 2014; Moscovitch et al., 2016; Goode et al., 2020). In addition to spatial navigation, it is likely that theta oscillations support memory functions by binding memory-related information from diverse neocortical structures to form episodic memories. Importantly, disruption of hippocampal theta coding impairs spatial processing and memory retrieval, demonstrating that theta oscillations play an indispensable role in hippocampus function (Shirvalkar et al., 2010; Petersen and Buzsáki, 2020).

### Gamma Oscillations

Gamma-band oscillations are fast rhythms (∼40–120 Hz) found in cortical-like structures in rodents, non-human primates, and humans (Buzsáki and Wang, 2012). Gamma oscillations arise from local synaptic inhibition and this is similar in the neocortex (Miltner et al., 1999; Whittington et al., 2011), hippocampus (Wang and Buzsáki, 1996; Csicsvari et al., 2003), and amygdala (Feng et al., 2019; Headley et al., 2021). One of the primary functions of gamma oscillations is thought to be the organization of cell assemblies (Buzsáki and Wang, 2012), a network of connected neurons that are synchronously activated by a particular cognitive process. Indeed, cell assemblies tend to be organized within gamma bursts such that individual assembly members are phase-locked to a preferential phase of gamma rhythms (Harris et al., 2003). Moreover, specific sub-bands of gamma rhythms likely have different underlying mechanisms and it is hypothesized that switching between various gamma modes may be an effective method of directing information flow (Pantev, 1995; Colgin et al., 2009; van der Meer, 2010; Ainsworth et al., 2011; Whittington et al., 2011; Colgin, 2015; Amir et al., 2018). For example, slow (∼25–55 Hz) and fast (∼60–100 Hz) gamma rhythms in the hippocampal CA1 region were found to synchronize with CA3 and the medial entorhinal cortex, respectively (Colgin et al., 2009). Moreover, slow and fast gamma rhythms appear to separately support spatial navigation and memory functions (Colgin, 2016). Interestingly, the amplitude of gamma oscillations tend to be modulated by the phase of the ongoing theta rhythm (Lisman and Jensen, 2013; Colgin, 2016). Theta-gamma coupling has been linked to learning and memory in rodents (Tort et al., 2009; Shirvalkar et al., 2010; Radiske et al., 2020), and abnormal theta-gamma coupling is associated with neurodegenerative disorders such as epilepsy and Alzheimer’s disease in humans (Kitchigina, 2018).

Collectively, theta and gamma oscillations, and their interaction, are intrinsically linked to hippocampal computations that subserve cognition function. Similar theta and gamma-range oscillations have been observed across many brain regions, suggesting that these oscillations are likely a common mechanism underlying neural coding across much of the mammalian brain (Headley and Paré, 2017). We may thus be able to extrapolate the function of hippocampal oscillations to the oscillatory activity commonly observed in other limbic regions, such as the amygdala and prefrontal cortex. Next, we will discuss how the interregional synchronization of oscillations facilitates adaptive anxiety-like behavior and fear regulation.

## Behavioral Paradigms for the Study of Fear and Anxiety in Rodents

Rodent models of anxiety-like behavior and learned fear have greatly contributed to uncovering the neural circuits underlying the regulation of emotions over the past several decades. Commonly employed behavioral paradigms for the investigation of anxiety-like behavior include the elevated plus maze (EPM) and open field (OF) assays. In the EPM, rodents are placed onto a novel elevated platform with four arms making a “+” shape (Walf and Frye, 2007). Two of the arms are enclosed by tall walls (closed arms) while the other two arms are open and exposed (open arms). This task pits the natural tendency of rodents to prefer protected areas against their innate motivation to explore novel environments. Similarly, in the OF task rodents are placed into a large open arena where the edges of the arena near the wall are interpreted as “safe” and the center is anxiogenic (Seibenhener and Wooten, 2015). Thus, an increase in time spent within the closed arm of the EPM or near the enclosing walls of the OF is interpreted as apprehensive or anxiety-like behavior (Bailey and Crawley, 2009).

Pavlovian fear conditioning has been the primary choice to study learned fear in both rodents and humans for many decades (Pavlov, 1927; Maren, 2001). In this procedure, an innocuous conditional stimulus (CS), such as an auditory tone, is paired with an aversive unconditional stimulus (US), such as a mild footshock. After a few pairings, animals develop fear to the auditory CS as it is predictive of the footshock US. In response to the CS (or conditioning context), rodents display freezing behavior (i.e., immobility), which is as an evolutionarily conserved defensive response to threat. Conditioned freezing to the CS can also be extinguished by repeatedly presenting the CS without footshock reinforcement, a process known as extinction (Bouton et al., 2021). Extinction does not erase the fear memory (i.e., CS-US association), but rather creates a new inhibitory memory that competes with the original fear memory (Quirk and Mueller, 2008). Pavlovian fear conditioning and extinction have direct clinical relevance as the circuits that underly adaptive fear learning are thought to be disordered in patients with anxiety- and trauma-based disorders, such as post-traumatic stress order (Ressler et al., 2022). Moreover, extinction learning is the basis for commonly used cognitive behavioral therapies, such as exposure therapy (Milad and Quirk, 2012; VanElzakker et al., 2014; Ressler et al., 2022).

## Hippocampal→Prefrontal Theta Transmission Underlies Anxiety-Like Behavior

Neural oscillations are not only critical to organizing cell assemblies locally but have long been proposed to facilitate long-range communication by synchronizing neural activity across brain regions. This hypothesis of “communication through coherence” is now widely accepted (Fries, 2005, 2015; Akam and Kullmann, 2012) (though see Schneider et al., 2021). In short, it is thought that neural oscillations facilitate interregional communication by creating temporally aligned windows of optimal neuronal excitability between sender-receiver systems. It is worth noting that theta rhythms observed in extra-hippocampal regions are often volume-conducted from the hippocampus (Sirota et al., 2008; Buzsáki et al., 2012; Lalla et al., 2017). That is, theta rhythms recorded in the prefrontal cortex or striatum, for example, may not be generated locally. That being said, this does not exclude the possibility of local theta generation in these regions nor does it undermine their functional relevance as hippocampal theta often acts to entrain local neural firing in extra-hippocampal regions (Buzsáki et al., 2012).

### The Dorsal and Ventral Hippocampus Are Functionally Distinct

The hippocampus can be divided into distinct dorsal (dHPC) and ventral (vHPC) subregions (posterior and anterior in humans, respectively) that are functionally and anatomically distinct (Fanselow and Dong, 2010). Although the entire hippocampus encodes spatial representations, the receptive fields of place cells within the hippocampus expand along the dorsoventral axis such that the dHPC encodes environments at a higher resolution than the vHPC (Kjelstrup et al., 2008). Moreover, the dHPC encodes highly precise spatial information irrespective of positive or negative motivational valence, whereas the vHPC more strongly represents non-spatial information (Jung et al., 1994). For example, Royer et al. (2010) found that, although vHPC neurons do not form continuous spatial representations like the dHPC, they do differentiate open and closed arms of a radial maze and display similar encoding of reward locations despite varying reward locations and trajectories. Oscillations in the dHPC and vHPC are similar and often tightly correlated, however they can diverge under a range of conditions (Schmidt et al., 2013; Sosa et al., 2020). In addition, the vHPC is the primary route by which the hippocampus projects to the amygdala (McDonald and Mott, 2017), ventral striatum (Friedman et al., 2002), and medial prefrontal cortex (Hoover and Vertes, 2007).

### Amygdala Inputs to the Ventral Hippocampus Transmit Motivationally Relevant Information

The vHPC receives dense inputs from the amygdala that likely contribute to the binding of spatial information with motivational salience (Yang and Wang, 2017). The amygdala plays a crucial role in processing both fear and reward and has thus been proposed to play a role of assigning emotional valence to motivationally relevant stimuli. In line with this, a subset of neurons in the basolateral complex of the amygdala (BLA) increases firing in the anxiogenic areas of the OF and EPM tasks (Wang et al., 2011). In addition, optogenetic excitation and inhibition of BLA→vHPC projections increases and reduces, respectively, anxiety-like behavior (Tye et al., 2011; Felix-Ortiz et al., 2013; Yang et al., 2016). Moreover, BLA theta preferentially synchronizes with the vHPC during states of anxiety (Jacinto et al., 2016). Although these findings may suggest that BLA→vHPC interactions preferentially drive anxiogenic states, other work shows that a subset of molecularly defined BLA→vHPC neurons exert anxiolytic effects (Pi et al., 2020), suggesting that BLA cell-type heterogeneity may underlie the differential transmission of positive and negative valence to the vHPC for emotional regulation and motivated navigation.

### The Hippocampus Transmits Negatively Valanced Spatial Information to the Prefrontal Cortex

Because hippocampal projections to the mPFC primarily arise from the vHPC, we might expect the mPFC to preferentially synchronize with vHPC oscillations. Indeed, researchers studying anxiety-like behavior using the EPM and OF discovered that the mPFC and vHPC (but not dHPC) display synchronized theta oscillations during exploration. Importantly, mPFC-HPC synchronization increases in the open arms of the EPM and the center area of OF (Adhikari et al., 2010). Remarkably, they found that the firing patterns of individual neurons in the mPFC become entrained to the phase of vHPC theta oscillations (Adhikari et al., 2010). In particular, theta-entrained mPFC neurons showed stronger modulation in the open arms of the EPM (Adhikari et al., 2011). Changes in vHPC theta oscillations are also predictive of changes in mPFC theta oscillations, suggestive of a vHPC→mPFC directionality that mirrors the underlying dense projections from vHPC to the mPFC (Adhikari et al., 2010). Optogenetic inhibition of the vHPC→mPFC projection disrupted mPFC unit-entrainment and produced an anxiolytic effect in the EPM and OF (Padilla-Coreano et al., 2016). This work suggests that vHPC theta synchronization preferentially conveys motivationally relevant spatial information to the mPFC and this may regulate navigation in potentially dangerous environments.

Although this work suggests that oscillatory coupling between the vHPC and mPFC is critical for anxiety-related behavior, it is unknown if vHPC theta oscillations play a causal role. Padilla-Coreano et al. (2019) elegantly addressed this question by testing if theta-paced (8 Hz), sine-wave stimulation of vHPC→mPFC projections was superior to standard square-wave (non-theta-paced) stimulation using optogenetics. They found that 8 Hz, but not 2 or 20 Hz, oscillatory stimulation of vHPC-mPFC pathways facilitated neural transmission, increased theta synchrony between vHPC and mPFC, and decreased time spent in the anxiogenic open arms (Padilla-Coreano et al., 2019). This work provides strong evidence that interregional theta synchrony enables long-range neural communication.

The vHPC does not uniformly transmit information to all downstream structures but instead selectively sends information *via* distinct channels to appropriate receiver networks. Ciocchi et al. (2015) recorded optogenetically identified vHPC neurons projecting to either the mPFC, amygdala, or the striatum during EPM, OF, and goal-directed navigation. Like previous work, they found that the anxiety-like behavior in the EPM and OF was encoded by mPFC-projecting neurons, whereas rewarded locations in a goal-directed navigation task were encoded by striatal-projecting neurons. Although they did not examine the relationship of striatal neurons to neural oscillations, others have shown that they are preferentially entrained to vHPC theta oscillations during goal-directed behaviors (Goodroe et al., 2018). This selective routing of information transmission is likely achieved by local microcircuitry within the vHPC that form functionally distinct motifs (Krook-Magnuson et al., 2012; Lee et al., 2014). Interestingly, amygdala-projecting neurons were not found to preferentially encode either appetitive or aversive spatial information (Ciocchi et al., 2015; Jimenez et al., 2018). This raises the question of what information this population does encode? Given the role of the amygdala in the storage of appetitive and aversive memories, we speculate that this pathway may preferentially act to engage BLA networks during the encoding and retrieval of context-dependent fear (Maren and Fanselow, 1995; Bazelot et al., 2015; Kim and Cho, 2017, 2020).

## Theta-Range Oscillations in the Encoding, Retrieval, and Expression of Fear Memories

### Amygdala Synchronization Enables Fear Memory Retrieval

Over the past three decades, there has been considerable progress understanding the role for network oscillations in aversive learning and memory. It was first discovered that neurons in the lateral amygdala (LA) display intrinsic theta rhythms after Pavlovian fear conditioning (Paré et al., 1995; Pape and Driesang, 1998; Pape et al., 1998). These rhythms were hypothesized to synchronize with the HPC to facilitate the retrieval of fear memories (Paré et al., 1995). It was subsequently shown that indeed the LA and dHPC synchronize at theta frequencies (4–12 Hz) during the retrieval of fear memories after both cued and contextual fear conditioning (Seidenbecher et al., 2003). One potentially confounding factor in this study was the different behavioral states of rats in the conditioned (freezing) and non-conditioned (locomoting) groups. It was therefore unclear if synchronization reflected memory retrieval or behavioral performance. However, in a subsequent experiment the investigators only observed LA-dHPC synchronization 24 h after conditioning, but not minutes to hours after conditioning, despite similar levels of freezing and theta activity at these time points (Pape et al., 2005; Narayanan et al., 2007b). Subsequent work in rodents has shown that amygdala theta also synchronizes with the mPFC during fear memory retrieval (Lesting et al., 2011; Likhtik et al., 2014; Stujenske et al., 2014; Davis et al., 2017; Ozawa et al., 2020), and similar findings have recently been observed using intracranial recordings in both humans (Zheng et al., 2019; Chen et al., 2021) and non-human primates (Taub et al., 2018).

Many decades of work have established that fear memories are initially encoded by a network of brain regions, including the amygdala, hippocampus, and prefrontal cortex (Maren and Quirk, 2004; Kim and Jung, 2006; Orsini and Maren, 2012; Herry and Johansen, 2014). Although the amygdala maintains long-term fear memory storage (Kim and Davis, 1993; Maren and Fanselow, 1996; Gale et al., 2004; Liu et al., 2022), the standard model of systems consolidation of memory posits that as memories age, their storage and retrieval become independent of hippocampal activity (Frankland and Bontempi, 2005). If true, this suggests the amygdala theta synchronization should shift to regions that are responsible for storing remote fear memories over time. In line with this, Narayanan et al. (2007a,b) found that LA-dHPC theta synchrony is not observed at remote timepoints despite similar freezing levels. However, LA-dHPC synchrony can be reinstated merely by context re-exposure, which is interpreted as a reflection of systems level memory reconsolidation (Narayanan et al., 2007a,b).

Indeed, it appears that the brain regions mediating fear memories shift over time, and the precise structures encoding these memories likely depends on sensory modality underlying the association. For example, Sacco and Sacchetti (2010) found that lesions of the secondary auditory, visual, and olfactory cortices impaired the recall of remote (but not recent) auditory, visual, and olfactory conditioned stimuli, respectively (Sacco and Sacchetti, 2010). These manipulations were modality specific, insofar as lesioning one of the sensory areas (i.e., the piriform cortex governing olfactory memory) did not affect memories of other modalities (i.e., auditory or visual) (Sacco and Sacchetti, 2010). Expanding on this, the authors showed that the BLA and secondary auditory cortex synchronize at theta frequency during the retrieval of remote, but not recent, auditory fear memories (Cambiaghi et al., 2016, 2017). Although memories may become less dependent on the HPC over time, there are instances where the HPC is still recruited at remote time points. For example, the remote retrieval of contextual fear memories is associated with increased synchrony between the hippocampus and anterior cingulate cortex (Wirt and Hyman, 2017, 2019; Makino et al., 2019). Collectively, these data reveal that theta synchrony accurately reflects memory retrieval processes across memory age and transformations.

### Respiratory-Related Oscillations Entrain Limbic-Wide Networks to Enable Fear Expression

Many of the early investigations into prefrontal and amygdala theta oscillations underlying aversively motivated behavior did not consider the possibility of distinct sub-bands of theta, but instead holistically grouped theta rhythms as 4–12 Hz. Recent work has now shown that respiratory-related rhythms exist within this frequency range and have functional significance to motivated behaviors. Respiratory-related oscillations (RROs) are slow rhythms that accompany breathing. These oscillations were discovered seven decades ago when Edgar Douglas Adrian found that LFPs in the olfactory cortex of hedgehogs and cats are synchronized to breathing patterns (Adrian, 1942). It is now clear that respiration organizes network dynamics across many limbic structures (Karalis and Sirota, 2022). Breathing patterns change with a variety of emotions (Homma and Masaoka, 2008), however it was only recently discovered that RROs are a major oscillatory correlate of fear expression in rodents (Tort et al., 2018a; Folschweiller and Sauer, 2021). Importantly, RROs and theta rhythms are dissociable, particularly during fear expression (Nguyen Chi et al., 2016; Tort et al., 2018b; Srikanth et al., 2021). For example, locomotor-related theta oscillations of the hippocampus typically occur with a peak frequency of ∼8 Hz, depending on movement speed and acceleration. However, fear-related immobility (i.e., freezing behavior) is tightly correlated to a prominent ∼4 Hz rhythm observed in the mPFC and BLA (Karalis et al., 2016). Although breathing frequency in rodents can range anyway from ∼1 to 15 Hz, they tend to breath at a frequency of approximately four inhalation/exhalation cycles per second (i.e., 4 Hz) during freezing behavior. This 4-Hz breathing pattern results in a synchronized 4-Hz oscillation in the olfactory bulb which then acts to entrain mPFC neurons for the maintenance of fear expression (Bagur et al., 2021). More specifically, 4-Hz RROs act to organize mPFC neurons into functional cell assemblies (Dejean et al., 2016) that likely control freezing behavior by synchronizing with the BLA (Karalis et al., 2016). Given the clear distinction of these rhythms from locomotor-related hippocampal theta oscillations, we will classify 3–6 and 6–12 Hz oscillations in the PFC and amygdala as RROs and theta oscillations, respectively.

The majority of work investigating oscillatory dynamics in aversive learning and memory has used Pavlovian fear conditioning procedures that elicit high levels of freezing behavior as the primary behavior output of fear. However, it should be noted that there is a wide range of fear-related behavior other than freezing (Gruene et al., 2015; Fadok et al., 2017; Mobbs et al., 2020; Jercog et al., 2021; Totty et al., 2021). Considering that locomotion is associated with 8-Hz theta rhythms in the hippocampus and amygdala, we might predict that active forms of fear expression, such as active avoidance, flight, or escape, might also be associated with 8-Hz theta rhythms, compared to freezing-related 4-Hz RROs. Indeed, a recent study using an active avoidance paradigm found that successful avoidance behavior was associated with increased 8-Hz theta power and reduced 4-Hz power, whereas unsuccessful avoidance trials dominated by freezing behavior instead showed increase 4-Hz power (Jercog et al., 2021). Similarly, Dupin et al. (2019) found that escape-like behavior during an odor fear conditioning paradigm were associated with increased 8-Hz theta power in the mPFC, BLA, and olfactory piriform cortex compared to freezing epochs. Although this suggests that 8-Hz theta rhythms are involved in the expression of active fear behaviors, it is currently unclear how these rhythms might differentially organize networks driving mobile fear states vs. mobile, non-fear-related states, such as exploratory behavior.

### Intrinsic Resonance Enables Selective, Pathway-Specific Information Routing

Given the stark contrast between fear related RROs and theta, it appears likely that ∼4-Hz RROs and ∼8-Hz theta rhythms represent orthogonal networks underlying these opposing behavioral states. We speculate that fear memory retrieval acts in part to reactivate these oscillatory network states. Evidence for this comes from a recent investigation into the relationship between memory engrams and oscillations in the BLA. Because parvalbumin-expressing (PV+) interneurons are heavily implicated in generating network oscillations, Davis et al. (2017) expressed “designer receptor exclusively activated by designer drugs” (DREADDs) exclusively in BLA PV+ interneurons that originally encoded fear memory formation (i.e., engram cells). Reactivation of PV+ BLA fear engrams increased freezing and drove ∼3–6 Hz BLA rhythms, whereas inactivation of this ensemble decreased freezing and drove ∼6–12 Hz rhythms (Davis et al., 2017). They further showed that distinct populations of BLA neurons display 4- or 8-Hz resonance in an experience-dependent fashion. Consistent with this, optogenetic stimulation of the BLA at 4 vs. 8-Hz bidirectionally regulated freezing behavior. Moreover, applying simultaneous 4-Hz stimulation to the mPFC and BLA increased freezing behavior only if this stimulation was in-phase (Ozawa et al., 2020). These findings support the notion that 4-Hz RROs and 8-Hz theta rhythms act to engage distinct BLA ensembles, and aligns with other work suggesting that positive- and negative-valance are encoded by independent subsets of BLA neurons (Paton et al., 2006; Belova et al., 2007; Redondo et al., 2014; Gore et al., 2015; Namburi et al., 2015; Beyeler et al., 2016, 2018; Kim et al., 2016; Lee et al., 2017; O’Neill et al., 2018; Zhang et al., 2020).

Given that fear and non-fear-related behaviors are driven by distinct anatomical pathways, it is possible that the resonance of BLA neurons may be predicted by their downstream projection targets. Amir et al. (2018) recorded BLA activity during a naturalistic foraging task and found high-gamma (75–95 Hz) was associated with apprehensive behavior and preferentially entrained neurons projecting to the mPFC over the NAc, which aligns with other work demonstrating increased high-gamma synchrony during fear retrieval (Stujenske et al., 2014). Moreover, it is also interesting to consider that hippocampal locomotor-related Type-1 (7–12 Hz) and fear-related Type-2 (4–7 Hz) theta appear to be analogous to BLA theta and RROs. We speculate that changes in HPC-BLA synchrony between Type-1 and Type-2 theta may be an efficient means for the context-dependent retrieval of fear memories (Maren et al., 2013). Future work should investigate these possibilities.

## Gamma Oscillations in Aversive Learning and Memory

Gamma oscillations have long been implicated in emotional learning and memory (Miltner et al., 1999; Keil et al., 2007; Headley and Paré, 2013; Luther et al., 2022). One of the first discoveries linking gamma rhythms to associative learning came when Miltner et al. (1999) found that ∼20–70 Hz gamma rhythms developed across occipital and parietal electrode sites in a Pavlovian conditioning procedure in humans. Interestingly, gamma coherence developed between visual and pericentral cortices, which represent CS and US information, respectively. This demonstrates an oscillatory correlate associative memory (Miltner et al., 1999). Similar to the hippocampus, gamma oscillations in neocortical and other cortical-like regions are thought to play a role in synchronizing groups of functionally connected neurons *via* precise periods of inhibition and excitation (Buzsáki and Wang, 2012; Sohal, 2016).

In the amygdala, gamma rhythms are generated by reciprocal interactions between inhibitory interneurons and excitatory pyramidal cells (Randall et al., 2011; Feng et al., 2019; Headley et al., 2021), and are most prominent in the basolateral (BL) nucleus, rather than LA, likely due to higher numbers of PV+ interneurons in BL (Headley et al., 2021). Gamma oscillations in the amygdala are associated with a wide range of behaviors and cognitive process. In relation to aversive learning and memory, gamma rhythms develop across conditioning (Bauer et al., 2007; Popescu et al., 2009; Courtin et al., 2014; Stujenske et al., 2014), emerge with retrieval (Bauer et al., 2007; Courtin et al., 2014; Stujenske et al., 2014), and are predictive of persistent fear memory (Courtin et al., 2014). BLA gamma oscillations are also pronounced during states of increased vigilance (Amir et al., 2018) and the expression of conditioned responses (Headley et al., 2021). Interestingly, a recent report found that activation of BLA PV+ interneurons *via* norepinephrine drives fear expression and reduces BLA gamma power in the BLA (Fu et al., 2022). This is in line with previous reports show that the release of norepinephrine in the BLA drives freezing behavior (Giustino et al., 2020) and that freezing behavior is associated with decreased gamma power (Stujenske et al., 2014).

Using Pavlovian fear conditioning paradigms, Headley and Weinberger (2011) found oscillatory correlates of auditory fear conditioning in the primary auditory cortex of rats. Specifically, they found that the amplitude of conditioning-driven gamma-band activation predicted associative memory strength 24 h later (Headley and Weinberger, 2011) and that conditioning-induced receptive field plasticity increased phase-locking of auditory cortical neurons to gamma rhythms (Headley and Weinberger, 2013, 2011). This suggests that learning-induced plasticity increases gamma frequency resonance in neurons that encode the initial experience, and in turn would allow these neurons to more easily be entrained by gamma rhythms for future memory recall. Expanding on this, the primary auditory cortex (Concina et al., 2018) and amygdala (Stujenske et al., 2014, 2022) synchronize with the mPFC at gamma frequencies during successful auditory fear discrimination. Stujenske et al. (2014) found that fast gamma (70–120 Hz) power and synchrony in BLA-mPFC-vHPC networks underly auditory fear discrimination, and that fast gamma oscillations are negatively correlated to freezing behavior. They further found that BLA fast gamma power increased with extinction learning and was associated with a mPFC→BLA lead, suggesting that fast gamma rhythms in the amygdala may reflect a safety-related signal (Stujenske et al., 2014). Conversely, it appears that slow gamma (40–70 Hz) rhythms in the amygdala may be associated with high fear and poor retention of extinction. Unfortunately, there is relatively little work examining gamma oscillations in the mPFC and HPC during aversive learning and memory which should be a focus of future investigations.

Gamma oscillations are also involved in memory consolidation (Huff et al., 2013; Kanta et al., 2019). In an eloquent study, Kanta et al. (2019) detected and modulated endogenous gamma oscillations in the amygdala using closed-loop optogenetics to probe their role in the consolidation of inhibitory avoidance memory. They found that boosting or diminishing gamma oscillations following learning significantly enhanced or impaired subsequent memory strength the next day, demonstrating a causal role of BLA gamma oscillations in the consolidation of contextual-based fear memories (Kanta et al., 2019). In summary, gamma oscillations are inextricably linked to a wide range of behaviors and cognitive functions in aversive learning and memory (Headley and Paré, 2013). Future investigations should focus on causal manipulations to determine specific functions of gamma oscillations across brain structures.

## Prefrontal Top-Down Theta Synchrony Regulates Fear Extinction

After fear conditioning, repeated presentations of the CS without the US will lead to extinction, which is manifest as a reduction in conditioned fear responses. During this procedure, animals learn that the CS no longer predicts the occurrence of the US. Interestingly, the suppression of fear is labile, and animals show a return of conditioned fear under a variety of conditions (Bouton et al., 2021). This reveals that extinction learning does not result in the erasure of the original fear memory but instead forms a new memory trace that competes with and inhibits the fear memory. Importantly, extinction learning is the basis of commonly employed cognitive-behavioral therapies for reducing fear and anxiety in anxiety- and trauma-related disorders (Milad and Quirk, 2012; Ressler et al., 2022). Although the oscillatory correlates of conditioned fear have been well described, much less is known about the role for oscillations in extinction learning and retrieval (Trenado et al., 2018).

Extensive work suggests that the prelimbic (PL) and infralimbic (IL) subregions of the mPFC are functionally dissociable such that the PL enables fear expression and the IL is critical for extinction (Laurent and Westbrook, 2009; Peters et al., 2010; Sierra-Mercado et al., 2011; Do-Monte et al., 2015; Giustino and Maren, 2015; Bloodgood et al., 2018). The IL has extensive connections with both the hippocampus and amygdala, two brain areas that exhibit oscillatory entrainment during Pavlovian fear conditioning. To explore the role for this network in extinction, Lesting et al. (2011) recorded from the LA, dHPC, and IL across fear conditioning and extinction. They found that the LA-dHPC-IL network displayed theta (4–12 Hz) synchrony early in extinction training (high fear) but displayed low synchrony late in extinction (low fear). The following day, IL-LA and IL-dHPC, but not LA-dHPC, showed high theta synchrony during extinction retrieval (low fear). Interestingly, these results hold true even when cross-correlation analyses were restricted to freezing epochs during extinction retrieval (Lesting et al., 2011). It is currently unclear if the rhythms observed in this experiment were 3–6 Hz RROs or 6–12 Hz theta rhythms. However, considering that the retrieval of extinction memories is associated with low levels of freezing we might speculate that mPFC-HPC oscillations underlying extinction retrieval are likely to be 6–12 Hz theta rhythms.

In subsequent work, Lesting et al. (2013) showed that the LA-dHPC-IL network displays no lead-lag relationship during fear memory retrieval, but the IL leads both the LA and dHPC during extinction retrieval. This mirrors anatomical work showing that extinction recall is associated with IL projections to LA, whereas fear recall is associated with prelimbic mPFC and hippocampal input (Knapska et al., 2012). This work reveals that the retrieval of fear extinction is associated with top-down theta synchrony such that mPFC leads dHPC theta. Although it has long been assumed that the mPFC does not project to the dHPC (Vertes et al., 2007), a recent report discovered for the first time that a population of mPFC long-range inhibitory neurons directly project to the dHPC and play a critical role in increasing hippocampal signal-to-noise ratio for spatial encoding by driving feedforward inhibition and increasing mPFC-dHPC gamma synchrony (Malik et al., 2022). Conversely, the mPFC has been proposed to interface with the dHPC *via* a disynaptic circuit through the nucleus reuniens of the thalamus (Vertes et al., 2007; Jin and Maren, 2015), which is also critical to fear extinction (Ramanathan et al., 2018a,b).

### Does the Ventral Midline Thalamus Enable Fear Extinction by Synchronizing Prefrontal-Hippocampal Theta Interactions?

The midline thalamus, comprised of in part the paraventricular nucleus (PVT), and nucleus reuniens (RE), has gained recent attention as key node in emotional regulation (Vertes et al., 2015; Dolleman-van der Weel et al., 2019; Cassel et al., 2021; Penzo and Gao, 2021). The PVT is suggested to guide adaptive selection of both positive and negatively motivated behaviors *via* projections to the nucleus accumbens and amygdala (Vertes et al., 2015; Choi and McNally, 2017; Penzo and Gao, 2021), whereas the RE is critical for navigation, spatial working memory, and episodic memory (Dolleman-van der Weel et al., 2019) *via* bidirectional connections with the hippocampus and prefrontal cortex (Vertes et al., 2007). It is widely believed that the RE is a critical node facilitating mPFC→HPC communication by serving as a hub in a mPFC-RE-HPC circuit (Xu and Südhof, 2013; Jin and Maren, 2015; Ferraris et al., 2018; Ito et al., 2018; Ramanathan et al., 2018a,b; Dolleman-van der Weel et al., 2019; Lin et al., 2020; Robinson and Brandon, 2021). Indeed, the RE (Ramanathan et al., 2018a; Silva et al., 2021) and incoming mPFC afferent neurons were recently shown to critical to the retrieval of extinction memories (Ramanathan et al., 2018a,b; Silva et al., 2021).

Given the extensive literature implicating mPFC-HPC theta in episodic memory processes (Eichenbaum, 2017), it has also been hypothesized that the RE may facilitate mPFC-HPC communication by synchronizing oscillatory activity in this network (Angulo-Garcia et al., 2018; Ferraris et al., 2018; Hauer et al., 2019, 2021; Cassel et al., 2021; Robinson and Brandon, 2021). For example, Hallock et al. (2016) found that pharmacological inactivation of the RE resulted in impaired mPFC-HPC coherence and working memory performance. They specifically found that mPFC→HPC directionality was impaired when animals entered the choice point of the T-maze task, a decision point that is known to be mPFC-dependent (Hallock et al., 2016). However, it is currently unclear how the RE mediates mPFC-HPC theta synchrony. It seems unlikely that the RE increases mPFC-HPC synchrony by directly driving theta and gamma rhythms, because RE inactivation selectively impairs mPFC oscillations, leaving HPC theta and gamma rhythms intact (Ferraris et al., 2018). Instead, it appears that the RE acts to coordinate the timing of synaptic and oscillatory events in the mPFC and HPC. For example, Ferraris et al. (2018) found that chemogenetic inactivation of the RE abolished mPFC-HPC gamma burst synchrony in anesthetized rats. It is also important to note that upstream regions that project to the mPFC, RE, and HPC may play a critical role in synchronizing all three structures. It was recently shown that the supramammillary nucleus (SUM) of the hypothalamus, a key structure in the generation and pace-making of hippocampal theta rhythms, is also critical for the transfer of information through the mPFC→RE→HPC circuit (Ito et al., 2018). Specifically, these authors found that SUM inactivation decreases the coherence of mPFC and RE neurons to CA1 theta at the decision point of a T-maze task (Ito et al., 2018). Although the RE was shown to be necessary for mPFC-HPC synchrony, this work suggests that synchronization of the entire mPFC-RE-HPC circuit may be coordinated by the SUM. Although we are currently unaware of any studies examining the potential involvement of the SUM in fear extinction, this seems likely given its role in coordinating mPFC→HPC theta oscillations *via* the RE.

## Conclusion

In summary, neural synchronization is an intrinsic aspect of neural computation that underlies various cognitive functions and behavior. Prefrontal theta synchronization *via* the vHPC mediates increases anxiety-like behavior *via* the transmission of motivationally salient spatial information. The retrieval of both recent and remote fear memories is characterized by theta and/or RRO synchronization with the amygdala, and the synchronized regions shifts with memory age. Oscillatory correlates of fear extinction match anatomical data suggesting that the mPFC provides top-down control of fear memories encoded in the amygdala and HPC. Although the mPFC does not project directly to the HPC, the nucleus reuniens of the thalamus appears to be a critical node in synchronizing prefrontal-hippocampal theta interactions for fear suppression.

## Author Contributions

MT and SM wrote the manuscript. Both authors contributed to the article and approved the submitted version.

## Conflict of Interest

The authors declare that the research was conducted in the absence of any commercial or financial relationships that could be construed as a potential conflict of interest.

## Publisher’s Note

All claims expressed in this article are solely those of the authors and do not necessarily represent those of their affiliated organizations, or those of the publisher, the editors and the reviewers. Any product that may be evaluated in this article, or claim that may be made by its manufacturer, is not guaranteed or endorsed by the publisher.
